# Stretching Method-Based Damage Detection Using Neural Networks

**DOI:** 10.3390/s22030830

**Published:** 2022-01-22

**Authors:** Emmanouil Daskalakis, Christos G. Panagiotopoulos, Chrysoula Tsogka

**Affiliations:** 1 Department of Mathematics, Vancouver Community College, 1155 E Broadway, Vancouver, BC V5T 4V5, Canada; edaskalakis@vcc.ca; 2 Department of Music Technology and Acoustics, Hellenic Mediterranean University, GR-74100 Rethymno, Greece; pchr@hmu.gr; 3 Department of Applied Mathematics, University of California, Merced, 5200 North Lake Road, Merced, CA 95343, USA

**Keywords:** machine learning, stretching method, damage detection

## Abstract

We present in this paper a framework for damage detection and localization using neural networks. The data we use to train the network are m×d pixel images consisting of measurements of the relative variations of *m* natural frequencies of the structure under monitoring over a period of *d*-days. To measure the relative variations of the natural frequencies, we use the stretching method, which allows us to obtain reliable measurements amidst fluctuations induced by environmental factors such as temperature variations. We show that even by monitoring a single natural frequency over a few days, accurate damage detection can be achieved. The accuracy for damage detection significantly improves when a small number of natural frequencies is monitored instead of a single one. More importantly, monitoring multiple natural frequencies allows for damage localization provided that the network can be trained for both healthy and damaged scenarios. This is feasible under the assumption that damage occurs at a finite number of damage-prone locations. Several results obtained with numerically simulated data illustrate the effectiveness of the proposed approach.

## 1. Introduction

The necessity and significance of Structural Health Monitoring (SHM) to ensure the long-term integrity of structures is well recognized. Damage inevitably occurs during the lifetime of a structure. It may be induced by environmental factors, operational conditions or a hazard event and it may cause premature aging, operation malfunction or even the failure of the structure. Monitoring structural damage is therefore considered to be extremely important for sustaining the original functionality and possibly extending the lifetime of structures.

To monitor structural health conditions, SHM systems have been implemented in almost all areas of engineering including civil, mechanical and aerospace engineering applications. In this context, SHM is defined as the process of detecting, localizing and possibly quantifying structural damage. Here, we think of damage as modifications that have occurred in an engineering structure affecting its performance with respect to some reference configuration, usually defined as the healthy state. An important category of SHM techniques is the vibration-based method, where the vibration response of a structure is recorded and analyzed using signal processing methods. A standard classification for vibration-based SHM procedures is that of parametric (response-based) and non-parametric (model-based) methodologies [1]. The latter category usually requires a numerical model for the structure, which may be a data-driven simulation of the existing structure. A recent review on vibration-based damage identification for civil engineering structures can be found in [2].

Our approach also falls into this class of vibration-based damage identification methods. The key idea of these techniques is that the damage-induced changes in the physical properties of the system will cause a detectable change in modal properties [3]. It is well known, however, that vibration-based damage identification methods are affected by environmental and operational variability [4]. This is because such variations also induce changes in modal properties. The main challenge is therefore to develop methods that can detect the effect of a small damage in the presence of noise induced by environmental variations.

With the recent explosion of machine and deep learning techniques, their use in SHM and related damage detection problems is also becoming very popular. We refer the reader to [5,6] for recent reviews focused on the use of machine learning and deep learning in SHM. A usual distinction between damage detection algorithms, which may be considered as anomaly detection [7], is into supervised, semi-supervised and unsupervised categories. The first one requires data of both the baseline and damaged (anomalous) configurations, while the second one needs only data for the baseline healthy configuration. In the latter case, there is no need for labeling the data. A supervised non-parametric approach is proposed in [8], while auto-associative neural networks are employed in [9] to discriminate system changes. In [10], a multilayer artificial neural network, which resembles an auto-associative neural network using temperature variables in addition to the frequencies as inputs, is explored to identify patterns in frequencies of undamaged structures under varying temperatures. An unsupervised machine learning approach is applied in [11] using PCA and an autoencoder using as inputs mode shapes and temperature in addition to natural frequencies for effective damage detection. Finally, more integrated approaches for long-term vibration-based SHM systems with real-time damage identification capabilities are proposed in [12] as well as in [13].

In this work, we only use eigenfrequencies as inputs, which we refer to as natural modes. The main source of noise is assumed to be induced by ambient temperature variations. Eigenfrequencies are much easier to be measured compared to the eigenvectors; i.e., the modal shapes of the structure under investigation. Specifically, we use the stretching method (SM) to estimate relative shifts in the natural frequencies (Δν/ν with ν denoting one of the eigenfrequencies of the structure) as in [14]. In these works, damage identification is performed by visually observing the graph of Δν/ν over time, looking for permanent and environmental noise-independent variations. We use SM because it was shown to provide a robust estimation of damage-induced variations in the natural frequencies amidst noise due to environmental variations such as wind, humidity and temperature.

The main contribution of this paper is the automation of the damage detection process using machine learning. More precisely, we propose to train a machine learning network to classify if a given measurement Δν/ν contains damage or not. In the proposed approach, Δν/ν measurements are considered as images, which we call a measurement image matrix (MIM). A MIM is of size m×d, with *m* denoting the number of natural modes monitored and *d* denoting the number of days over which we monitor the variations of Δν/ν. These MIMs are used to train different neural networks that have the ability to detect damage or even localize a damage. Several configurations are considered using single or multiple natural frequencies monitored over a single day or a multiple day period. In general, the results improve as the number of natural frequencies monitored and the number of days over which they are monitored increases. Typically, several networks can be trained and used in parallel. This allows for both early damage detection using single-day MIMs as well as localization using multiple-day and/or multiple-mode MIMs. Note that the SHM problem has long been perceived as fundamental in statistical pattern recognition [15,16].

Training such a network requires a large set of measurements of Δν/ν corresponding to a structure with and without damage; in contrast, for localization, measurements corresponding to different damage locations are needed. For that, we assume that damage usually occurs in structures at a finite number of damage-prone locations. Then, we can produce such data utilizing numerical models; for example, a finite element-based model of the structure. Note that the idea of digital twins, containing detailed and simplified models as well as components of co-simulation entities through the dynamic decoupling of the structure [17], has already been employed; for example, in [18]. Thus, obtaining such measurements seems feasible.

The remainder of the paper is as follows. In Section 2, we briefly present SM and explain how we use it to compute Δν/ν from which MIMs are formed. In Section 3, we describe the neural network we use for damage detection, while in Section 4, we explain the process that we use to generate realistic MIMs for both damaged and healthy scenarios accounting for ambient temperature variations. In Section 5, we present results for damage detection and localization using MIMs to train neural networks. Finally, Section 6 contains our conclusions.

## 2. The Stretching Method for SHM

**Figure 1:**
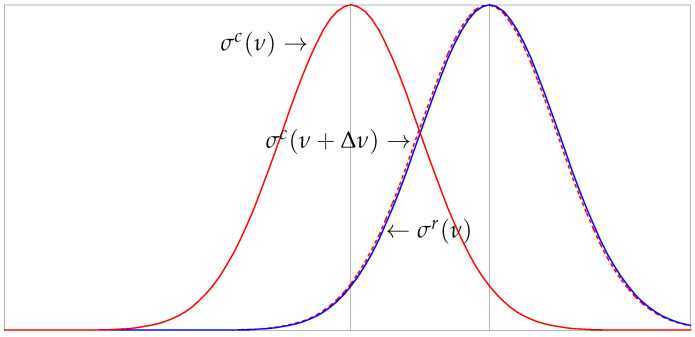
Stretching of function σ(ν) for ν defined in some specific range in order to fit with $\sigma^{r}\left(\nu \right)$, Δν is the maximizer of correlation coefficient $C_{s}$ given in Equation (1).

The stretching method is a signal processing technique widely used in geophysics to detect velocity variations in the Earth’s interior [19,20,21]. More recently, this method was used to monitor natural frequency variations in the context of SHM [14,22]. The idea is to monitor changes in the natural frequencies of a structure by estimating a frequency shift Δν that maximizes the normalized correlation coefficient [14]

(1) $$C_{s}\left(\Delta \nu \right)=\frac{\int_{\nu_{1}}^{\nu_{2}}\sigma^{c}\left(\nu +\Delta \nu \right)\sigma^{r}\left(\nu \right)d\nu }{\sqrt{\int_{\nu_{1}}^{\nu_{2}}{\left(\sigma^{c}\left(\nu +\Delta \nu \right)\right)}^{2}d\nu \int_{\nu_{1}}^{\nu_{2}}{\left(\sigma^{r}\left(\nu \right)\right)}^{2}d\nu }}\;.$$

where σ(ν) is understood as an appropriate mode indication function [23]. The basic assumption is that this frequency-dependent quantity, σ(ν), undergoes a constant shift Δν in the frequency range $\left[\nu_{1},\nu_{2}\right]$, as is also shown in Figure 1. In other words, we assume that the current form of the frequency quantity, denoted as $\sigma^{c}$, may be approximated as $\sigma^{c}\left(\nu +\Delta \nu \right)$ ≈ $\sigma^{r}\left(\nu \right)$ for ν in the range $\left[\nu_{1},\nu_{2}\right]$, with $\sigma^{r}$ being the reference quantity. If there is no frequency shift between the reference and the current quantity, then the minimizer of Equation (1) is Δν=0, as expected.

The idea is to work with a quantity σ that is a function of the natural modes of a structure. In practice, we proceed as follows: assuming *n* degrees of freedom are measured, $u^{i}$, i=1,…,n (e.g., acceleration measurements along different directions at different locations), we compute the empirical cross-correlation of these measurements over the time interval [0,T],

(2) $$C_{T}^{i,j}\left(\tau \right)=\frac{1}{T}\int_{0}^{T}u^{i}\left(t\right)u^{j}\left(t+\tau \right)dt,$$

with τ denoting the lag-time. Using different time intervals [0,T], we compute two quantities: the reference one for which *T* is of the order of days and the current one for which *T* is of the order of hours. Then, we form the matrix

$${\left[A^{\#}\left(\nu \right)\right]}_{i,j}=A_{i,j}^{\#}\left(\nu \right),\;\;\#=r,\;c$$

where $A_{i,j}^{\#}\left(\nu \right)$ is the Fourier transform of the reference (#=r) or current (#=c) empirical cross-correlation defined in Equation (2),

(3) $$A_{i,j}^{\#}\left(\nu \right)=\int C_{T}^{i,j}\left(\tau \right)e^{ı2\pi \tau \nu }d\tau ,$$

for 1≤i,j≤n. By construction, the matrix $A^{\#}\left(\nu \right)$ is symmetric, and thus it admits a symmetric singular value decomposition (SVD) of the form,

(4) $$A^{\#}\left(\nu \right)=U^{\#}\left(\nu \right)\Sigma^{\#}\left(\nu \right)U^{{}^{\#},T}\left(\nu \right).$$

The matrix $\Sigma^{\#}\left(\nu \right)$ is a real diagonal matrix with the singular values $\sigma_{1}^{\#}\left(\nu \right),\ldots \sigma_{n}^{\#}\left(\nu \right)$ placed on the diagonal while the columns $u_{1}^{\#}\left(\nu \right),\ldots ,u_{n}^{\#}\left(\nu \right)$ of the matrix $U^{\#}\left(\nu \right)$ are the corresponding singular vectors. Here, $U^{\#},T$ denotes the transpose of the matrix $U^{\#}$. The frequencies at which the first singular value admits peaks correspond to the natural frequencies of the structure, while the first singular vector at the corresponding frequency is identified as the modal shape associated to this natural frequency (see Section 10.3.3 of [24]).

To monitor changes in the natural frequencies of a structure, we can use Equation (1) with σ equal to $\sigma_{1}\left(\nu \right)$, the larger singular value of the matrix A(ν). Alternatively, we can also use the Fourier transform of the cross-correlations, $A_{i,j}^{\#}\left(\nu \right)$, directly. As we know from [14], all the peaks present at the first few singular values are also present in $A_{i,j}^{\#}\left(\nu \right)$. Moreover, as observed in [14], working with $A_{i,j}^{\#}\left(\nu \right)$ directly presents some advantages: (i) the cost of the singular value decomposition is avoided and (ii) more accurate measurements of Δν/ν are obtained. The latter is true because not all modes appear in all the $A_{i,j}^{\#}\left(\nu \right)$ components. Thus, it is possible to decouple modes that correspond to nearby frequencies by examining different $A_{i,j}^{\#}\left(\nu \right)$ components.

Note that by adjusting the frequency window $\left[\nu_{1},\nu_{2}\right]$ in Equation (1), the shifts for several natural modes of the structure can be monitored. Traditionally, the identification of a damage is performed by visually observing the graph of Δν/ν over time looking for permanent and temperature-independent shifts. The measurements of Δν/ν may be regarded as an image (i.e., each element of the matrix may be viewed as a pixel in the image), which we call the measurements image matrix (MIM). The dimension of the image is m×d, with *m* denoting the number of natural modes monitored and *d* the number of days over which they are monitored. Using these MIMs as data, a machine learning network is trained to classify if a given MIM contains damage or not. Moreover, as we show in Section 5.2, not only damage detection but damage localization may be also accomplished by monitoring only a few natural frequencies.

This requires a large set of Δν/ν measurements for both the healthy and the damaged structure; in contrast, for localization, measurements corresponding to different damage locations are needed. Measurements of Δν/ν without damage can be easily obtained from the structure’s normal state, but measurements for a wide range of damage states are realistically not available. To avoid this pitfall, we produce data here using a simplified finite element model of the structure, which in specific cases and under certain assumptions may be representative for some real structure; for example, the masonry lighthouse in Rethymno’s port [22]. This finite element model is used to produce data for both damaged and undamaged scenarios. The SM method is subsequently used to produce Δν/ν measurements from which the MIMs are formed.

## 3. Classification Using Neural Networks

In this section, we describe the proposed methodology for identifying potential damage by replacing the visual inspection of Δν/ν measurements with a machine learning algorithm. As explained in Section 2, using SM, we obtain Δν/ν measurements. These measurements are used to train a simple neural network [25]. Neural networks have been successfully used to classify an image library into different classes depending on the subjects of those pictures. In our application, the images of different subjects are replaced by the MIMs which are m×d pixel images. In the training set, half of the MIMs contain a damage that occurs on the fifth day and the other half correspond to a healthy configuration with no damage. The neural network is trained to classify the MIMs into two classes: “MIMs with damage” and “MIMs without damage”.

**Figure 2:**
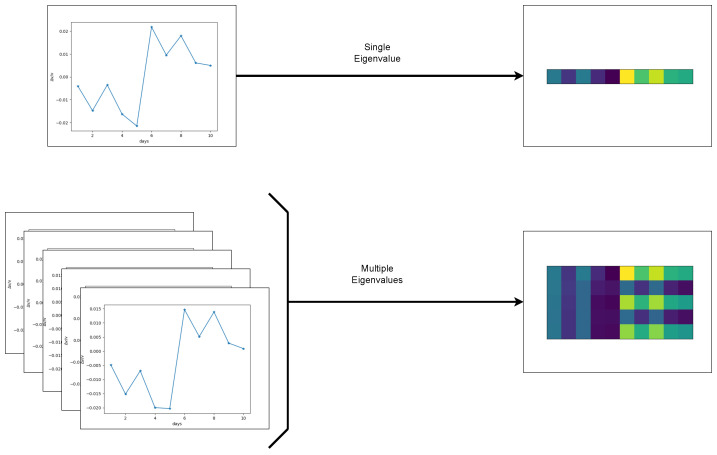
For the purpose of this work, Δν/ν measurements are used to form the MIMs which are images of m×d pixels. Here, *m* denotes the number of natural modes monitored and *d* the number of days over which they are monitored. Here, we illustrate MIMs using either one (**top**) or five (**bottom**) natural modes monitored over a 10 day period.

As illustrated in Figure 2, a single Δν/ν measurement corresponding to one natural mode may be considered as a one-dimensional image. We will see that considering multiple Δν/ν measurements corresponding to multiple natural modes significantly improves the performance of the method. In this case, the measurements may be regarded as two-dimensional images. For the heatmaps (right column) in Figure 2 and elsewhere in the paper, we do not show the colormap. This is because the actual values do not play an important role, the key observation being that we need to recognize the pattern that is created in the presence of damage.

The neural network that we use consists of a single hidden layer, the size of which is determined by the size of the input data; i.e., it depends on how many natural mode measurements are used to construct the MIMs. The first layer of the neural network is a flattened layer—a non-learning layer that acts as a transformation of the 2D MIMs to a 1D array. Then, we have one hidden layer—a multiple connected layer whose dimension is md×2, with md the dimension of the input and 2 the dimension of the output. The last layer is the output layer and has two possible outcomes: “damage” or “no damage”.

To train the network, we use 80% of the available synthetic data, and the remaining 20% are used to evaluate the efficacy and the efficiency of the classification network. After the training, if a test MIM is given, the trained network will return a probability of “damage” or “no damage”.

## 4. Data Acquisition for Training

**Figure 3:**
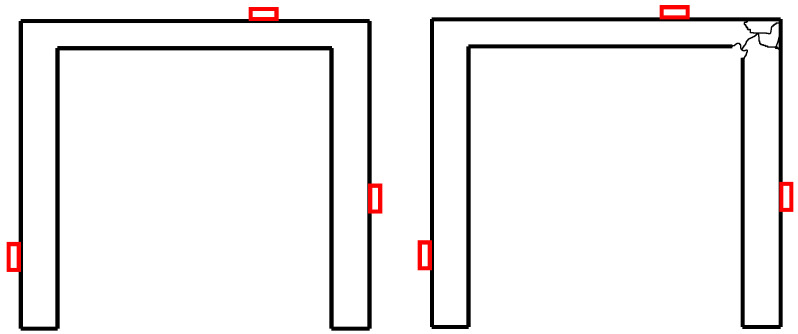
Schematic illustration of healthy and damaged structure. The red boxes indicate the sensors’ locations.

In this section, we present the data acquisition procedure. As mentioned earlier, data corresponding to a structure with and without damage are needed. The damage considered could be any local stiffness degradation of structural members; e.g., some joint deterioration in a concrete structure, as illustrated in the schematic example in Figure 3. In practice such data could be collected from a structure and/or its digital twin. To validate the method and illustrate its performance, we use in this paper synthetic, numerically simulated data generated with a finite element model of a simple structure for both damaged and undamaged scenarios.

The structure considered is a simply supported beam. Despite its simplicity, it allows us to obtain the necessary structural features. Furthermore, this structure can be considered as a standard benchmark system since it has been used in previous research; e.g., [10,11]. We focus on the effect of temperature on the mechanical properties of construction materials as the dominant environmental variable affecting the dynamic features [4].

### Raw Ambient Noise Data Acquisition

**Figure 4:**
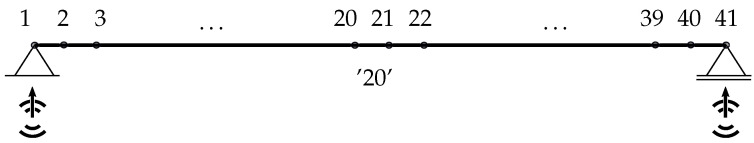
Sketch of the finite element model of the beam where some node numbers are indicatively shown, as well as the 20th element where damage is to be introduced.

Following previous research [11], the structure under investigation is a simply supported beam with length *L* = 5 m, cross section area $A=1.624\times 10^{-3}$$m^{2}$ and moment of inertia *I* = $1.971\times 10^{-6}$$m^{4}$. A value for the mass density equal to ϱ = $3693.5\;{\mathrm{kg}/m}^{3}$ is used so that the first natural frequency of the structure is close to 0.5 Hz for an ambient temperature of 15 °C. For the finite element model, an assembly of 40 beam elements has been considered as illustrated in Figure 4. The nonlinear relation between the material’s elasticity modulus *E* and the temperature *T* is assumed to be of the form

(5) $$\begin{array}{c} E=\left(206.216-0.4884T+0.0044T^{2}\right)\times 10^{9}{N/m}^{2}. \end{array}$$

where the temperature *T* is in Fahrenheit units.

Note that for reference, for the healthy configuration of the simply supported beam, the natural modes are known analytically:

(6) $$\begin{array}{c} \omega_{n}\left(T\right)\;=\;n^{2}\pi^{2}\sqrt{\frac{E\left(T\right)I}{\varrho AL^{4}}} \end{array}$$

Damage is considered by reducing the stiffness using a degradation coefficient that multiplies the moment of inertia of specific beam cross-sections. The smallest degradation coefficient leads to the highest damage level.

Using FEM, performed in a pure Java framework for numerical methods mainly developed by one of the authors (CGP) and freely available on the internet [26], the ambient noise recordings are simulated. Then, the empirical cross-correlations are computed using Equation (2) for *T* equal to 24 h. The computation of a $A_{i,j}^{\#}$ for a specific day, according to Equation (2), requires ambient noise recordings $u^{i}\left(t\right)$ for a duration of 24 h for the current quantity and 7 days for the reference. In practice, data are stored in smaller segments, so we proceed as follows: first, the cross-correlations of these smaller segments are computed; then, the average of all the cross-correlations corresponding to every given day is obtained, and then $A_{i,j}^{\#}\left(\nu \right)$ is computed by performing the Fourier transform on the average.

For our study case, we generate small segments of measurements corresponding to ambient temperatures from −15 to 45 degrees Celsius. To calculate the $A_{i,j}^{\#}\left(\nu \right)$ for a day for which, for example, the temperature varies from 5 to 10 degrees Celsius, we randomly select such small segments of simulated data obtained for the same temperature range. We use enough of those small segments so that the total length of the recordings used corresponds to one day of measurements.

**Figure 5:**
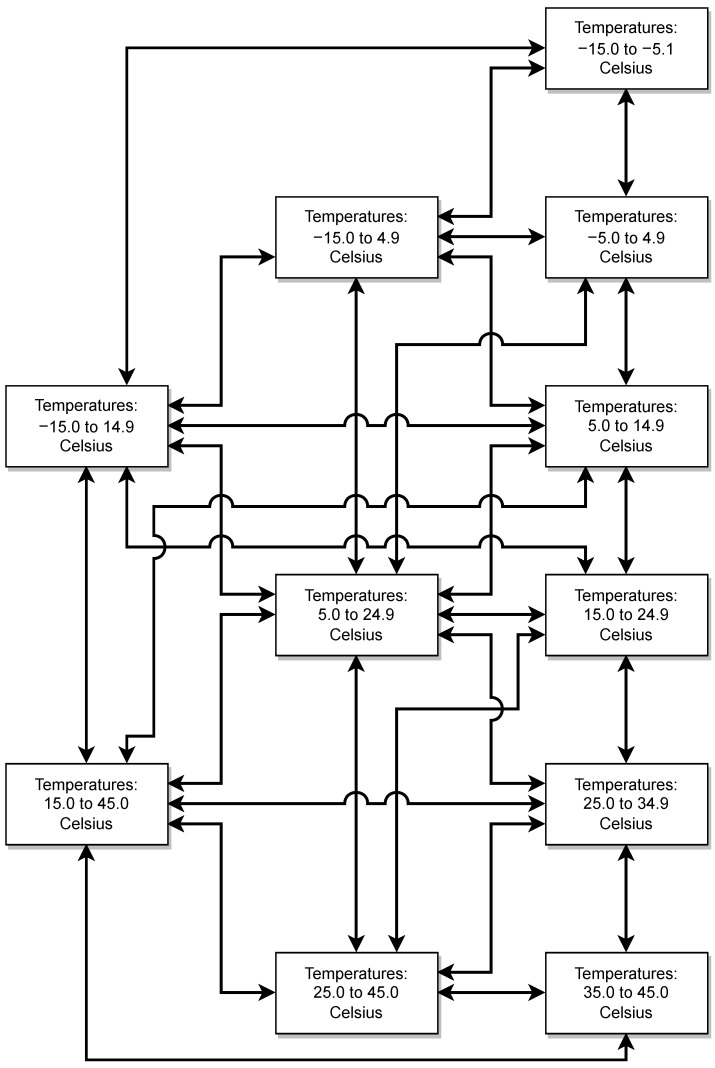
Temperature graph: To construct a *d*-day MIM, we chose data from different temperature groups for each day. The temperature group for the first day was chosen randomly, and the temperature groups for the following day were chosen according to the graph above.

For our training to form the MIMs, we need Δν/ν measurements for *d* consecutive days. To realistically consider day to day temperature variations, we randomly select the temperature variation range from predetermined temperature ranges, and then we semi-randomly select the temperature variation of the following day according to the undirected graph of Figure 5 to guarantee that day to day temperature variations are not too extreme too often. For example, it is not possible for one day that belongs to the temperature group −15 to −5 Celsius to be followed by a day that belongs to the temperature group 35 to 45 Celsius. Using the graph to generate the measurements allows for relatively large but realistic variations of temperatures within the *d* day measurement of Δν/ν. The data produced are challenging for the classification problem since large temperature variations could be misclassified as damage. We test different levels of damage in order to determine the size at which damage will no longer be differentiated from temperature variations.

Following the temperature selection process described above, we produced a large number of *d*-day long Δν/ν measurements.

## 5. Results

In this section, we show the results for damage detection and localization using MIMs to train our neural network. Our measurements have realistic temperature variations that affect the mechanical properties of the structure under investigation as described in Section 4. We start with the simple network that uses MIMs for a single natural mode and show that using machine learning indeed improves the accuracy of damage detection compared to the visual inspection of the Δν/ν curve. We next show that using MIMs for multiple natural modes significantly improves the accuracy of the network. Another advantage of using multiple natural modes is that a neural network can be trained beyond detection to damage localization as well.

### 5.1. Damage Detection

#### 5.1.1. Single Mode Neural Network

**Figure 6:**
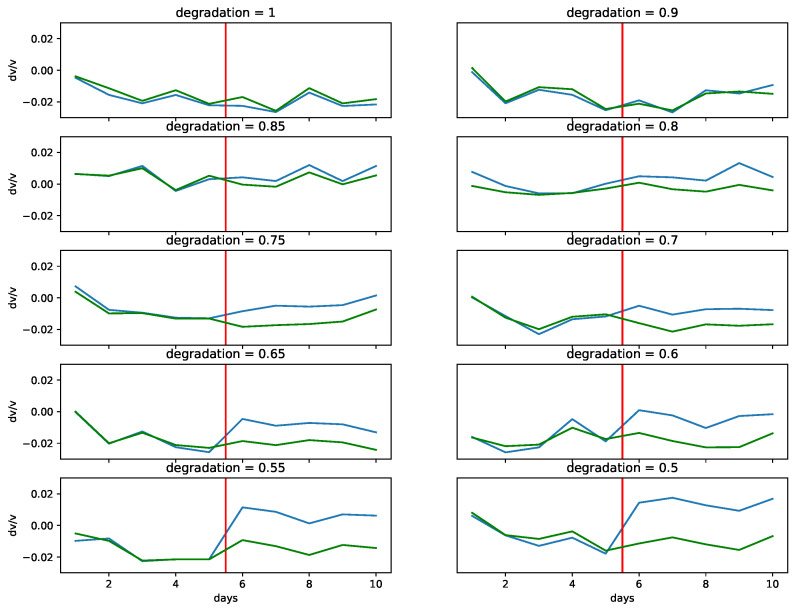
Examples of the first (green) and second (blue) natural modes for different degradation coefficient values. The red vertical line indicates the day on which the damage occurs. For the first natural mode, it becomes quite challenging to distinguish damage from variations due to temperature for examples with a degradation coefficient larger than 0.6. The second natural mode does not show any measurable variation in Δν/ν because the damage is located near one of the nodes of the second natural mode (see Figure 7—red curve).

**Figure 7:**
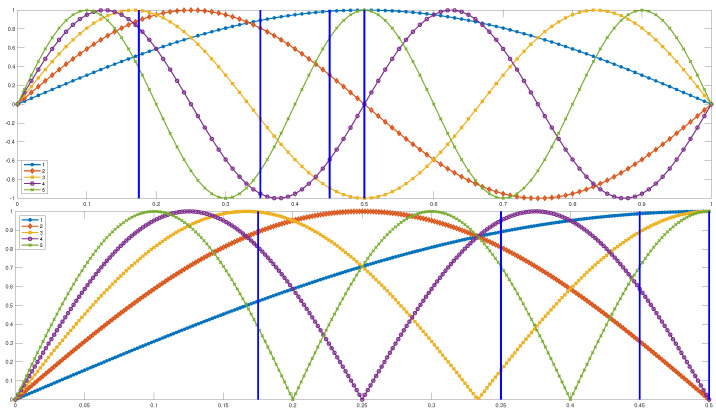
The shapes of the modes and the locations of the damage. The bottom plot considers the symmetry, and it is given in absolute values.

Following the process described in Section 4, we produce 10,000 MIMs with some level of damage determined by the degradation coefficient and another 10,000 MIMs without any damage. Here, the monitoring period is d=10 days long. Figure 6 shows some typical examples of measurements that correspond to the first two natural modes, for different levels of damage occurring at the middle of the 10 day period. For the first natural mode, we observe that as the degradation coefficient approaches 1 (smaller damage level), the jump of Δν/ν at the middle of the 10 day period becomes increasingly difficult to distinguish from temperature-induced variations. In general, for a degradation coefficient larger than 0.6, the damage becomes undistinguishable from temperature variations when we visually inspect the graph of Δν/ν. For the second natural mode, we do not observe any jump in the value of Δν/ν, and this is independent of the level of the damage. This is due to the location of the damage, which is very close to one of the nodes of the second natural mode (see Figure 7—red curve).

**Figure 8:**
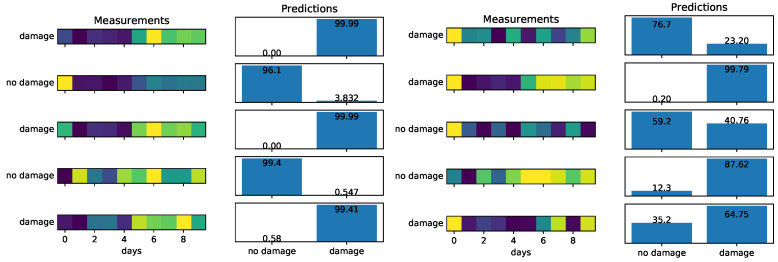
Performance of the proposed neural network using a single natural mode. On the left, the degradation coefficient is 0.5 (severe damage easy to be identified), and on the right, the degradation coefficient is 0.8 (limited damage that is hard to be identified).

**Figure 9:**
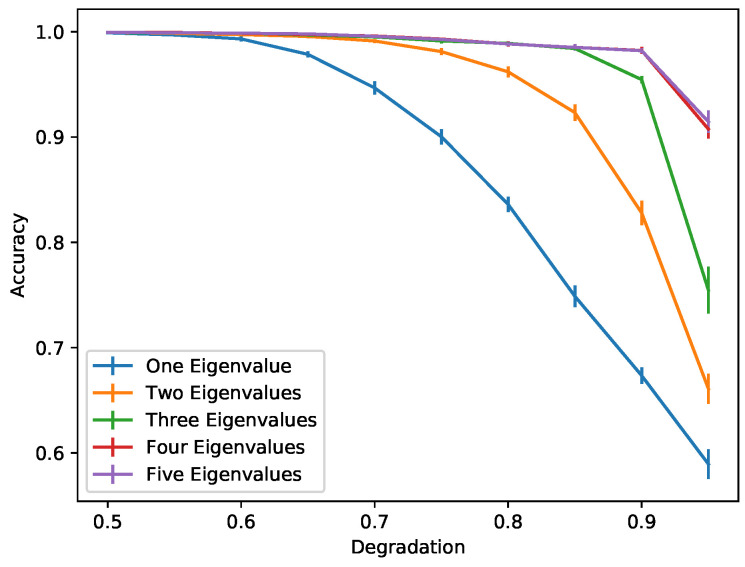
Comparison of the performance using different numbers of natural modes for different levels of damage.

Training a simple neural network using MIMs of the first natural mode slightly increases the chances of correctly identifying measurements containing damage as compared to visual inspection. As we can see in Figure 8, in the left column, when the degradation coefficient is 0.5, we correctly identify damage in all the examples considered. However, when the degradation increases to 0.8 (smaller damage; see Figure 8, right column) we may misidentify (first example) or correctly identify the damage but with high uncertainty (third and fifth example). The accuracy of the neural network for different degradation coefficients when using one natural frequency is given by the blue color plot in Figure 9. We see that an accuracy of at least 90% can be achieved for damage levels corresponding to a degradation coefficient of 0.75 or smaller.

#### 5.1.2. Multiple Mode Neural Network

As observed in Figure 6, the second natural mode does not show any measurable variation in Δν/ν due to the imposed damage. Even for the severe damage case when the degradation is 0.5 and the first natural mode clearly shows damage, the second natural mode does not show anything. This difference in behavior is due to the fact that the damage is located very close to a node of the second natural mode. Indeed, the damage is located at the middle of the beam, and as we can see in Figure 7, two out of the first five eigenmodes have a node at this location. This explains why we cannot measure the damage at this location using the second natural mode.

The observation above raises the question of what to expect if we use more than one natural mode. In that case, the measurements will look like 2D images where each row is the measurement of a natural mode and each column is a different day-measurement. In our case, we use the first five natural modes, so the MIM can be considered as a 5×10 pixel image. If we assume no damage, then we expect similar Δν/ν behavior for the five natural modes (every natural mode should have similar Δν/ν when this is only induced by temperature variations). On the other hand, when there is damage to be detected, there is going to be a difference between the natural modes depending on how close the nodes of the eigenmodes are to the location of the damage.

**Figure 10:**
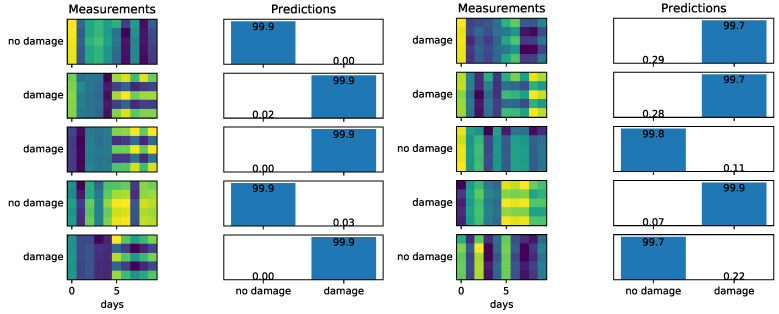
Performance of the proposed neural network using the first five natural modes. On the left, the degradation coefficient is 0.5 (easy case), and on the right, the degradation coefficient is 0.8 (hard case).

Indeed, as we can see in Figure 10, using five natural modes dramatically increases the accuracy of the neural network, since detecting a pattern created by the different behaviors of eigenmodes is a much easier task than trying to distinguish damage from temperature variations of a single eigenmode. In Figure 9, we can see how the accuracy of the neural network is improved by increasing the number of natural modes used. The accuracy in this case is measured as the average probability with which the trained network correctly identifies the damage status of the 20% of synthetic data used for testing the network. A neural network that uses four or five natural modes provides an accuracy very close to 1, which means that we can correctly identify damage even as the damage gets smaller; i.e., the degradation gets very close to 1.

Going from the single-mode to a multiple-mode neural network allows us to detect specific patterns after the damage has occurred. That is because different modes feel the damage in a different way depending on the location of the damage (see Section 5.2). The pattern that emerges after the occurrence of the damage helps us not only with the damage localization, as we see in the next section, but also allows us to detect the presence of damage using only single-day measurements. This is possible by comparing patterns created by measurements of single columns of the MIM.

**Figure 11:**
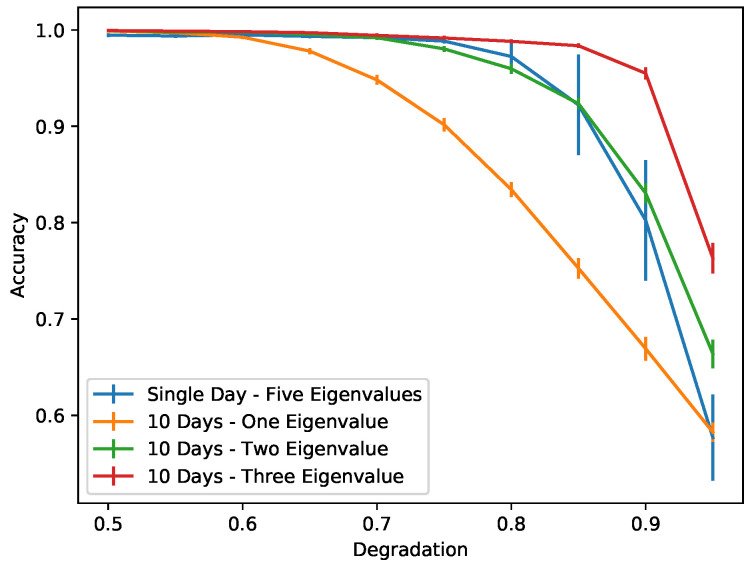
Comparison of the performance between networks using a single and a 10-day long MIM. A single-day MIM network performs similarly to a 10-day long MIM network that uses two natural modes.

To illustrate this, we train a new network using only one day as a healthy measurement and one day of unhealthy (damaged structure) measurement. In this case, damage detection is based on the pattern that is created, since for different modes we observe Δν/ν with different amplitudes. In Figure 11, we see the comparison of a single-day MIM network and a 10-day MIM network. A single-day network performs similarly to a 10-day network that uses the first two eigenvalues only. In Figure 9, we have observed better performance by using four or five eigenvalues in the 10-day measurement network for smaller damage levels. This means that a single-day network will be less reliable but also will require less time for measurements. This suggests that such a network is appropriate for crude immediate guesses of the probability of damage while waiting for more measurements that will provide a more accurate estimate.

**Figure 12:**
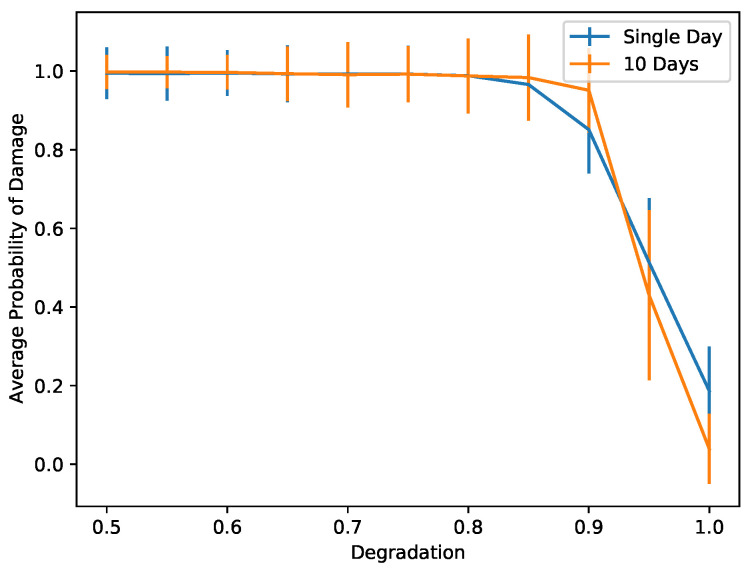
Comparison of the performance between networks using a single and a 10-day long MIM when the training is done with a degradation coefficient of 0.8 while the testing is performed for damages with different degradation levels. Five natural modes are monitored.

Another question that arises is the effect of the degradation level we use to train our network on how well we can detect a damage that occurs but has a different degradation level. In Figure 12, we can see that a network trained with a degradation of 0.8 can easily detect a larger damage but struggles if the damage level is lower than the one we used to train the network. As a result, we suggest to always train the network with a very low level of damage so that a broader range of damage level can be detected.

A direct qualitative comparison for similar examples given in the literature (see for example [10,11]) indicates the very good performance of the current approach. It is worth mentioning that while the current approach is a supervised one, it does not use temperature variations as an input of the network.

### 5.2. Damage Localization

We have already illustrated that using multiple natural modes significantly increases the accuracy of the neural network in identifying the presence of damage. The reason behind this improvement is that the neural network has in this case information about how well the damage is seen by the different natural modes. However, how the damage affects the different natural modes is determined by the location of the damaged which motivates us to modify our neural network to determine the location of the damage as well.

**Figure 13:**
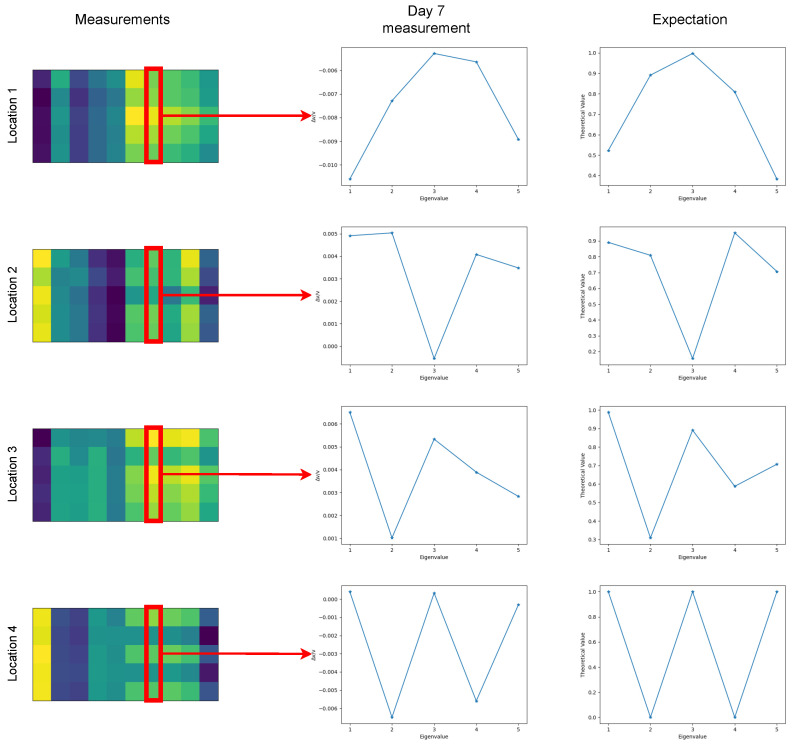
Examples of MIMs from the first five natural modes (first column), the measurement from the seventh day (second column) and the amplitudes of the corresponding modes at the location of the damage (third column). We can see that the pattern created because of the damage after the fifth day closely resembles the pattern of the modal amplitudes at the same location.

We assume that the appearance of damage will not tremendously affect the mode shapes. The main factor that determines how well a natural mode detects damage is the amplitude of the modal shape at the location of the damage. If the damage is located at a node (zero amplitude) of the modal shape, we expect the corresponding natural mode not to detect the damage. On the other hand, when the damage is located close to the maximum amplitude of the mode, then the corresponding natural mode will detect the damage with the maximum Δν/ν (see Figure 13).

In Figure 7, we can see the shapes of the modes for the first five natural modes for the finite element model we used. Due to the symmetry of the shape of modes, we are not able to uniquely determine the location of the damage (except for the middle of the beam), so we focus on the left half of the beam. Needless to say that for real world applications, actual symmetry in the modes is rare, and so focusing on the left part of the beam is not unreasonable.

We now consider three additional possible damage locations, making a total of four locations, and following the same procedures as before, we generate data for the neural network training using 0.8 as the degradation coefficient. This time, the task is to locate where the damage is. The idea here is that the MIMs will show a different pattern for the days that there is damage because of the fact that the shapes of the modes have different amplitudes at different damage locations. More specifically, in Figure 13, we can see examples of MIMs for the four locations (see locations at Figure 7). During the seventh day, which is a day on which damage has happened, we compare the corresponding Δν/ν measurements for the first five natural modes with the amplitude of the corresponding modes on the same locations. As we can see, the Δν/ν measurements closely match the amplitudes of the corresponding modes. This means that if there is damage, we expect to see a specific pattern for the days after day five, and the pattern will match the amplitude of the corresponding modes.

**Figure 14:**
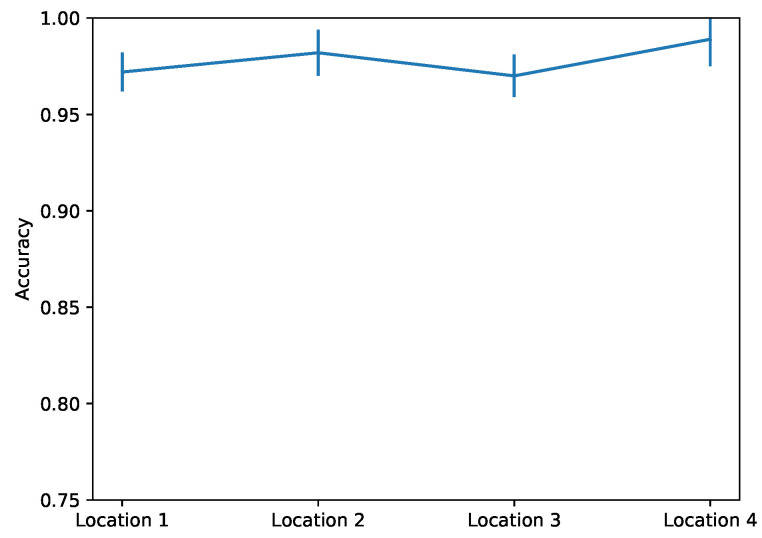
Accuracy for damage localization. MIMs monitoring five natural modes over a 10 day period are used.

This observation implies that a network can be trained to classify between the four different patterns that correspond to the four damage locations. In Figure 14, we give the accuracy of the network for the four damage locations, revealing an overall high accuracy in damage localization. This experiment involves only four different locations, but we expect similar accuracy even for more damage locations as long as the corresponding patterns differ. It should be pointed out that in the majority of real structures, we may consider that there is a finite number of such possible locations of damage appearance; for example, the joints of a reinforced concrete or steel frame.

**Figure 15:**
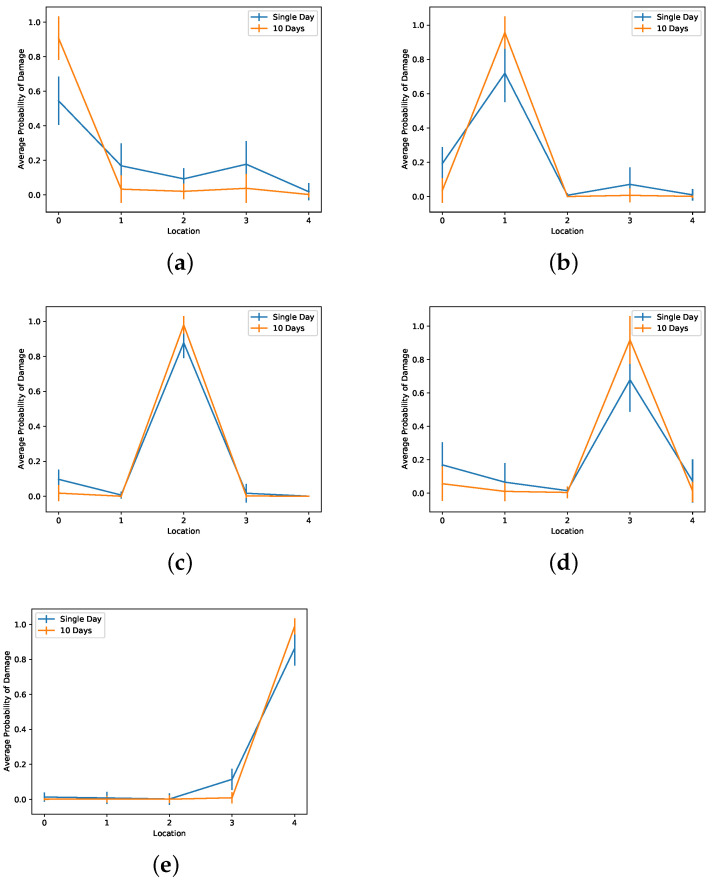
Average probability of damage obtained after training a network with five classes; measurements without damage (**a**) and measurements with damage at four locations (**b**–**e**) corresponding to locations 1 to 4 respectively. Location 0 means no damage. In general, we detect the location of the damage with a higher probability when we use multiple-day measurements instead of single-day measurements. Five natural modes are monitored.

A more practical test is training a network to classify damage between the four locations and the scenario without damage. To test the network, we ask for predictions using sets of test MIMs with damage in the four locations: a set for a specific location of the four possible ones and also MIMs without damage. In Figure 15, we can see the average probability of damage when we provide MIMs with damage at specific locations. In Figure 15a, we see the distribution of the average probability of damage among the different locations when using MIMs without damage. We observe that single-day MIMs have a higher probability of a false positive, identifying a MIM as one that comes from a structure with damage while that is not true. Using 10-day long MIMs, we avoid having false positives. On the other hand, as we see in Figure 15b–e, when we provide MIMs with damage, we are more likely to identify them as such.

At this point, we can observe that any neural network that uses the 10-day MIMs performs much better than a single-day MIM neural network. However, a 10-day MIM network will only be able to detect damage with a lag of several days in comparison to the single-day MIM networks that can detect damage within a single day. It is worth mentioning that the actual amount of days, for a multiple-day measurement, does not have to be 10. The optimal number of days may be different for different structures and can be determined using a sensitivity analysis. A system composed of several types of networks can provide both early detection results as well as more accurate results with a lag of several days. Such a system can be practically useful for an inspection team to begin preliminary inspection until the multiple-day MIM networks can provide more details about the existence and the location of the potential damage.

### 5.3. Additive Noise

All the results we have presented so far are based on simulated data. The simulated data lack any type of instrument noise that real world data could possibly have. To study the effect of instrument noise, we train our neural network using five natural mode measurements based on simulated noise-less data (degradation =0.8), as in the previous sections. Here, we test the network with noisy measurements produced by adding to the data Gaussian additive noise corresponding to different SNR values.

**Figure 16:**
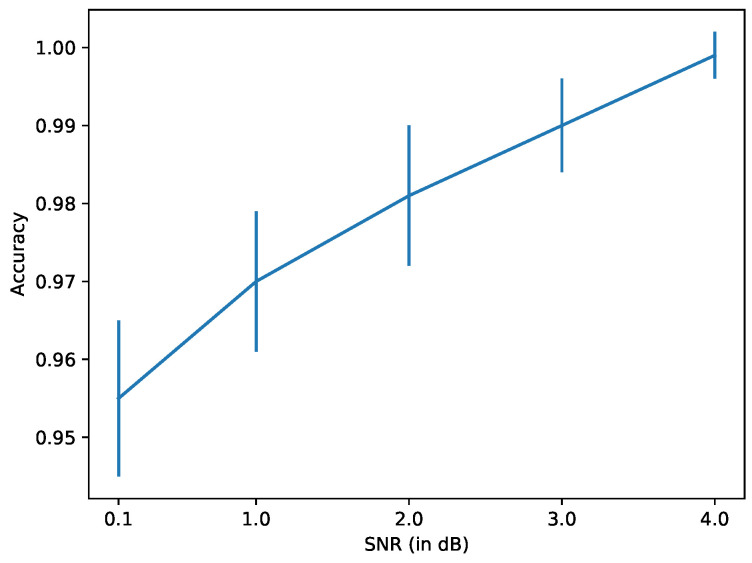
Neural network using MIMs monitoring five natural modes over a 10-day period. Accuracy of damage detection using noisy data with different SNR values.

We observe in Figure 16 that the accuracy is indeed affected as the noise in the data increases. However, for all the levels of noise tested, the accuracy remains above 0.95, which is quite high. This robustness to additive measurement noise is expected since the cross-correlation and averaging over time we perform in SM alleviates most of the effects of the noise.

The results presented here are very satisfactory and show the good performance of the proposed approach. Its robustness to noise suggests that the method can be easily applied to more realistic scenarios for structures. As expected, when the degradation coefficient increases, the damage becomes smaller and therefore becomes more difficult to detect. Increasing the number of observed natural frequencies is expected to improve the performance of the network. Furthermore, preliminary results indicate that the performance improves when temperature variation data are included in the input of the network.

## 6. Conclusions

In this paper, we presented a new framework using neural networks to automate the damage detection and localization procedure in Structural Health Monitoring applications. The network is trained with simulated data for both healthy and damaged scenarios. Our approach is based on the stretching method presented previously in the literature. A significant advantage of the proposed methodology is that only natural frequencies are used, which are much easier to measure compared to natural shapes. The data we feed the network comprise the measurement image matrix (MIM)—an m×d pixel image with *m* being the number of natural modes monitored over a period of *d*-days. Our results obtained using a simple structure and including realistic ambient temperature variations are very encouraging. They suggest that relatively small damages can be detected and localized by monitoring only a few natural modes. Networks trained with a single-day MIM can be used for early damage detection, while using multiple-day MIM significantly improves the accuracy and allows for damage localization. The effect of additive measurement noise was also studied, and the approach is shown to be stable to noise as very accurate results were obtained even for low signal-to-noise ratios.

In this work, we intentionally did not use the temperature variations as inputs, as this was not necessary for our supervised machine learning approach. Our next focus will be on unsupervised machine learning approaches. Preliminary results show that for such techniques to be effective, measured temperature variations in addition to frequencies should be considered as inputs. Other future research directions include the application of the proposed approach to experimental data from laboratory setups corresponding to more realistic complex structural systems.

## Data Availability

The data presented in this study are available on request from the corresponding author.
